# Morphology and genome size of *Epipactis helleborine* (L.) Crantz (Orchidaceae) growing in anthropogenic and natural habitats

**DOI:** 10.7717/peerj.5992

**Published:** 2018-12-20

**Authors:** Agnieszka Rewicz, Monika Rewers, Iwona Jędrzejczyk, Tomasz Rewicz, Jeremi Kołodziejek, Anna Jakubska-Busse

**Affiliations:** 1 Department of Geobotany and Plant Ecology, Faculty of Biology and Environmental Protection, University of Lodz, Łódź, Poland; 2 Department of Agricultural Biotechnology, UTP University of Science and Technology, Laboratory of Molecular Biology and Cytometry, Bydgoszcz, Poland; 3 Department of Invertebrate Zoology and Hydrobiology, Faculty of Biology and Environmental Protection, University of Lodz, Łódź, Poland; 4 Department of Botany, Institute of Environmental Biology, University of Wrocław, Wrocław, Poland

**Keywords:** *Epipactis helleborine*, Morphology, Genom size, Anthropogenic, Adaptation

## Abstract

**Background:**

The process of apophytism or spreading native species to human-made habitats is one of the main elements in the creation of plant cover in anthropogenic areas. Lately, an increase of anthropogenic localities with valuable flora has been observed. Apophytes are also members of the family Orchidaceae, especially from the genus *Epipactis*. The aim of the study was to (i) determine and compare the phenotypic variation of *E. helleborine* (L.) Crantz plants in anthropogenic and natural habitats, (ii) compare the genome size of plants growing in natural and anthropogenic habitats. The results reported in this study may indicate that a habitat influences morphological characteristics of plant species.

**Methods:**

Field studies were conducted on four native stands and four stands in anthropogenic areas of *E. helleborine* in Poland in years 2011–2013. Biometrical analyses were performed on shoots and flowers. The flowers were characterised by 25 biometric features and measured using a Nikon SMZ 800 binocular, microscopic Moticam-1SP cameras and the MIPlus07 programme (Conbest Co.). The nuclear DNA content was determined in fresh and young leaves of *E. helleborine*, collected from four natural and four anthropogenic populations.

**Results:**

We observed that in anthropogenic populations: (i) shoots were higher than shoots from natural populations, (ii) flowers differed significantly in terms of ten biometric features between habitats, (iii) the genome size of some population differed significantly between plants growing in natural and anthropogenic habitats.

**Discussion:**

According to some researchers, the presence of phenotypic variability and the occurrence of ecotypes are adaptation strategies of plants to environmental changes. In our opinion, in the case of the studied anthropogenic habitats (roadside) in which the *E. helleborine* populations grew, we can talk about ecofen due to the often repeated set of characteristic features, i.e., high shoots, long inflorescence and long, broad leaves. We agree, however, that it is difficult to isolate a taxonomic unit for ecofen due to the lack of experimental research.

## Introduction

The family Orchidaceae comprises 20,000 to 30,000 estimated species, making it the largest family of flowering plants (Baumann, Kunkele & Lorenz, 2010; Delforge, 2006; Pedersen & Mossberg, 2017). Orchids are considered to be ubiquitous, since they occur on all vegetated continents and even some Antarctic islands (Dressler, 1994). Their distribution and abundance vary between continents and regions; however, the most orchid-rich areas include South America, Madagascar, Sumatra and Borneo for mostly epiphytic species, Indochina for both epiphytic and terrestrial species, and Western Australia as a centre of terrestrial orchid richness (McCormick, Whigham & O’Neill, 2004). In Europe, there are approximately 130 species (Pedersen & Mossberg, 2017). Despite a great number of orchid species, many are rare or even threatened with extinction (Dressler, 1994). It is observed that some orchid species disappear in their natural habitats and penetrate anthropogenic environments (Dressler, 1981; Reinikka, 2008). The first report about the appearance of orchids in anthropogenic areas came from the 19th century, when those plants were observed at railway embankments in Great Britain (after Procházka & Velísek, 1983). *Dactylorhiza majalis* (Rchb.) P.F. Hunt & Summerh and *Epipactis helleborine* (L.) Crantz are species which the most frequently occupy anthropogenic habitats (Bîtea et al., 2011; Light & MacConaill, 2005; Light & MacConaill, 2006; Rewicz et al., 2017a; Wittig & Wittig, 2007). The genus *Epipactis* includes 15–80 species (Delforge, 2006; Jakubska-Busse et al., 2017; Kreutz & Fateryga, 2012; Pedersen & Mossberg, 2017) and systematics of this genus is complicated mainly due to similar morphological features. Jakubska-Busse (2008), Jakubska-Busse et al. (2016) have also observed some morphological adaptations to local environments in this genus. One of such adaptations is the change in floral architecture and the possibility of transition between cross- and self- pollination (Tałałaj & Brzosko, 2008). This genus has very asymmetric and very complex karyotypes, which causes a variation in the number of chromosomes between the *Epipactis* species during the differentiation process. Verlaque, Seidenbinder & Reynaud (1987) suggested that the basic chromosome number is *x* = 10.

*Epipactis helleborine* is a clonal taxon, growing in broadleaved, mixed and coniferous (also secondary) forests, on forest edges and also in anthropogenic habitats, such as rural and urban roadsides, railway embankments, post-mining sites, tracks, quarries, poplar plantations, parks, sandy beaches, lawns (Akhalkatsi, Arabuli & Lorenz, 2014; Hollingsworth & Dickson, 1997; Wittig & Wittig, 2007) and, furthermore, also in cities (Milović & Mitić, 2012; Rewicz et al., 2017a; Stešević & Jovanović, 2008). This species is rather indifferent in terms of habitat and behaves as a pioneer (Delforge, 2006). It grows on moderately wet, acidic to neutral humus soils and sometimes on substrates rich in calcium carbonate (Robatsch, 1983).

Observations of the authors as well as data from the literature suggest that in habitats changed by humans *E. helleborine* populations are characterised by high morphological variability of ramets. Moreover, higher and more massive shoots compared to the populations in natural habitats are observed in these populations, which can suggest possible differences in their genome size (Adamowski, Stefaniak & Święczkowska, 2012; Rogalska, Małuszyńska & Olszewska, 2005; Stefaniak et al., 2011). The somatic chromosome numbers reported for this species range from 2*n* = 20 for the diploid cytotype (Weijer, 1952) to 2*n* = 60 for the hexaploid cytotype (Averyanov, Averyanova & Lavrenko, 1982; Meili-Frei, 1965); however, other numbers, such as 2*n* = 36, 38, 39, 40, were also reported (Silvestre, 1983). Jakubska-Busse (2008), Jakubska-Busse et al. (2016) report that *E. helleborine* is a morphologically changeable species, which can be a result of several ecological factors or somatic mutations occurring in ramets within one genet. This species displays a wide range of phenotypic variability which allows it to more easily adapt to changes in the environment.

The family Orchidaceae is characterised by high levels of phenotypic plasticity. Heywood (1974) claims that the phenotype modification is a response of the genotype to the surrounding environment, where changes frequently occur on the genetic level, including changes in the genome size (Rogalska, Małuszyńska & Olszewska, 2005). Considering the genome size of the Orchidaceae, high variation is observed, with the genome size ranging 168-fold, from 0.66 to 110.8 pg/2C (Leitch et al., 2009). There is no doubt that the huge range of variation in DNA content has a significant effect on their phenotype. Therefore, the determination of C-value is an important feature for biology and biodiversity of the Orchidaceae (Bennett, Bhandol & Leitch, 2000). Earlier studies revealed a relationship between the genome size and latitude, altitude at sea level, temperature or precipitation, but there is no consensus as to whether the correlation is negative or positive (Bogunic et al., 2007; Knight & Ackerly, 2002). Vinogradov (2003) has indicated that the species with larger genomes possess less adaptability to adverse environmental conditions, and at the same time, the risk of their extinction is much higher than that of the species with small genomes. Within the orchid family polyploidy was also detected (Jacquemyn, Meer & Brys, 2001). Polyploids are characterised by a large size and vigour of cells, leaves, flowers, and fruits compared to diploid individuals (Tamayo-Ordóñeza et al., 2016). They are also more tolerant to changing environmental conditions and have more chance for expansion to new areas. This is probably related to an increased degree of heterozygosity, which can be an essential factor for growth, development and adaptability of polyploids (Tamayo-Ordóñeza et al., 2016). Since chromosomes of many orchids are small and often numerous, ploidy estimation by chromosome counts is difficult. In addition, microscopic chromosome counting is time-consuming and limited to a few tissues. Therefore, flow cytometry (FCM) is a more convenient alternative for establishing the ploidy/genome size of the Orchidaceae species. The genome size is, next to morphological descriptions, and molecular methods, good taxonomic marker useful for identifying many problematic taxa and phylogenetic reconstruction of closely related species (Ávila Diaz & Oyama, 2007; Fajardo, De Almeida Vieira & Molina, 2014; Rewers & Jedrzejczyk, 2016; Ståhlberg & Hedrén, 2008; Soliva, Kocyanb & Widmera, 2008; Szczęśniak, Gola & Jedrzejczyk, 2017; Wang et al., 2016). In the literature, we can find results, which show correlation between 2C DNA amount and morphological, ecological parameters of many groups of plants (Ahmadian et al., 2017; Knight, Molinari & Petrov, 2005; Knight & Ackerly, 2002). So we believe that in disturbed habitats such as anthropogenic habitats polyploids should occur more often and it is a result of adapting them to new conditions.

The objectives of this study were to: (i) determine and compare the phenotypic variation of the *E. helleborine* plants from anthropogenic and natural habitats, (ii) compare the genome size estimation of plants growing in natural and anthropogenic habitats.

## Material and Methods

### Study sites

**Figure 1:**
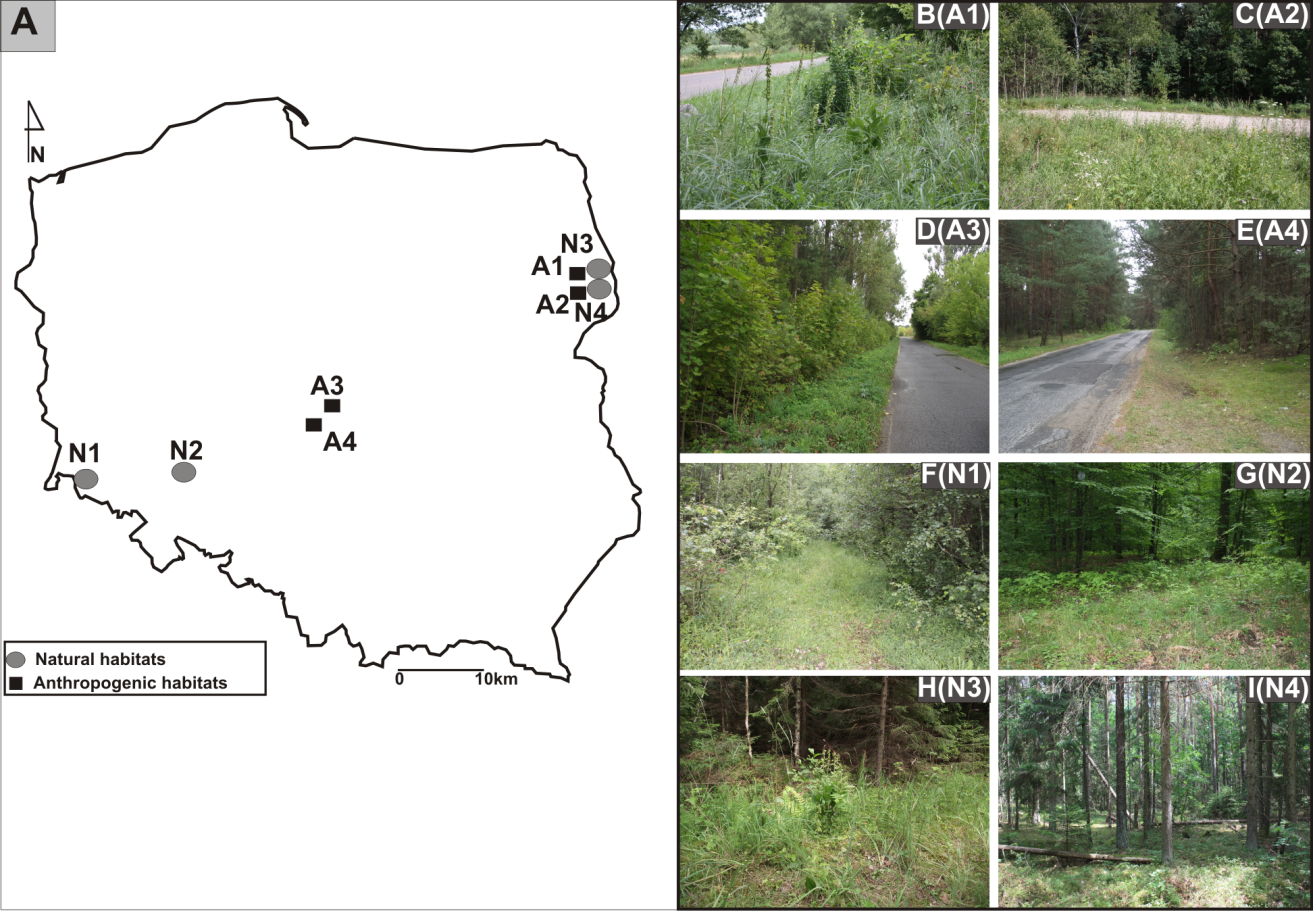
Distribution of studied populations of *E. helleborine*. (A) List of localities of the studied populations of *E. helleborine*: (B) A1—Guszczewina, (C) A2—Hajnówka, (D) A3—Sulejów 1, (E) A4—Sulejów 2, (F) N1—Kaczawskie Mt, (G) N2—Siechnice, (H) N3—Białowieża Primeval Forest 1, (I) N4—Białowieża Primeval Forest 2. (fot. A. Rewicz).

The research was carried out in Poland from 2011 to 2013. The study sites were located in three different geographical regions: from the Białowieża Primeval Forest, Northeast Poland, through Central Poland, to the Province of Lower Silesia, Southwest Poland (Figs. 1A, 1B-I).The identified investigated habitats were separated into two categories: the populations found in anthropogenic habitats such as roadsides and in natural habitats such as mixed forests (Table 1). Experimental studies and material sampling were done with the consent of the Regional Director for Environmental Protection (permit WPN6400.74.2013.MW).

**Table 1:** List of localities of the studied populations of *E. helleborine*. A1, Guszczewina; A2, Hajnówka, A3, Sulejów 1, A4, Sulejów 2, N1, Kaczawskie Mt, N2, Siechnice, N3, Białowieża Primeval Forest 1, N4, Białowieża Primeval Forest 2.

**Population code**	**Habitat type (Locality)**	**Populationsize (m**^2^**)**	**Number of shoots**	**GPS coordinates**
A1	roadside (Guszczewina)	36	127	N 52.831600 E 23.794836
A2	roadside (Hajnówka)	108	102	N 52.734217 E 23.603314
A3	roadside (Sulejów)	460	80	N 51.353793 E 19.883155
A4	roadside (Sulejów)	46	152	N 51.349757 E 19.882484
N1	mixed forest (Kotowice)	100	300	N 51.027997 E 17.239203
N2	mixed forest (Kaczawskie Mts)	40	150	N 51.041241 E 17.176701
N3	mixed forest (Białowieża Primeval Forest)	120	34	N 52.828706 E 23.797095
N4	mixed forest (Białowieża Primeval Forest)	400	41	N 52.832427 E 23.763069

### Biometric analysis

**Figure 2:**
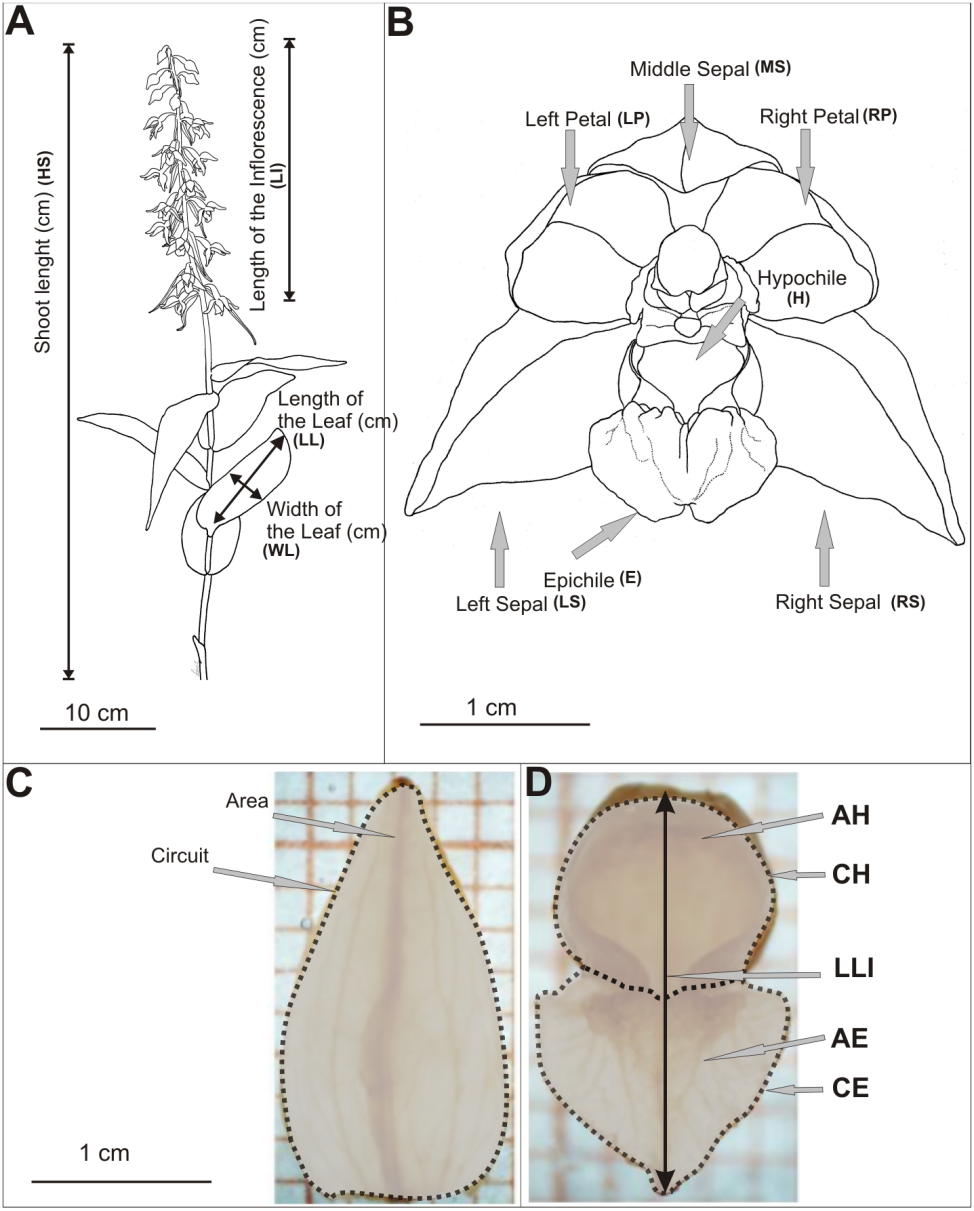
Diagram of measurements. (A) Shots of *E. helleborine*, (B) flower, (C–D) patterns (see the Table S1) (Figure A and B drawn by Z. Łobas).

Biometrical analyses were performed on shoots and flowers of *E. helleborine* (Table S1, Fig. 2A). The length of the shoot, the inflorescence and the length of leaf (the second from the bottom) were measured. We measured all shoots in the populations. Live measurements were taken using a measure tape rounded up to the nearest 1 mm. Study on the variability of metric features of *E. helleborine* flowers was carried out in August 2013, taking randomly a sample of 15 flowers from each population (second flower from the bottom were taken from randomly chosen 15 shoots). The flowers were inserted into the preservative Kew Mixture (composition for 1 litre: 530 ml 96% EtOH, 50 ml formaldehyde, 50 ml of glycerol, 370 ml of distilled water), which allowed to maintain the shape and natural size of flowers for further research. Each elements of flower was measured on a separate basis. The flowers were characterised by 25 biometric features (Table S1, Figs. 2B–2D) and measured using a Nikon SMZ 800 binocular, microscopic Moticam-1SP cameras and the MIPlus07 programme (Conbest Co.).

### Genome size estimation

The nuclear DNA content was determined in fresh and young leaves of *E. helleborine*, collected from eight populations. The leaves of *Secale cereale* ‘Dankowskie’ (2*C* = 16.2 pg) (Doležel & Bartoš, 2005) were used as an internal standard. The studied samples were prepared according to Jędrzejczyk & Śliwinska, (2010). The plant material was chopped with a sharp razor blade in a plastic Petri dish containing 1 ml of nucleus-isolation buffer (0.1 M Tris, 2.5 mM MgCl_2_ ×6H_2_O, 85 mM NaCl, 0.1% (v/v) Triton X-100; pH 7.0) supplemented with propidium iodide (PI, 50 µg/mL) and ribonuclease A (50 µg/mL). Nuclei suspension was passed through a 50 µm mesh nylon filter. For each sample, measurements of fluorescence intensities were performed in at least 7,000 nuclei using a CyFlow SL Green (Partec GmbH, Münster, Germany) flow cytometer equipped with a laser with green light emissions at 532 nm. Five individual plants were analysed per population. The measurements were performed at the same day and the samples were analyzed alternately. Histograms were analysed using FloMax software (Partec GmbH, Münster, Germany). Only histograms of high quality, with mean CVs under 5% for the target species, were used in the study. The nuclear genome size of *E. helleborine* was calculated using the linear relationship between the ratio of the target species and *S. cereale* 2C peak positions on the histogram of fluorescence intensities. The mean coefficients of variation (CV) of the 2C nuclei were estimated for all the samples of *E. helleborine*. The 2C DNA contents (pg) were transformed to megabase pairs of nucleotides, using the following conversion: 1 pg = 978 Mbp (Doležel & Bartoš, 2005).

### Statistical analysis

Biometric data were statistically analysed using STATISTICA ver. 10.0 and Canoco ver. 4.5. The following basic characteristics were calculated: the arithmetic mean (*x*), minimum and maximum value, standard deviation (SD) and coefficient of variation (CV). The compatibility of the studied morphological features with the standard distribution was checked by means of the Shapiro–Wilk and Kolmogorov–Smirnov tests. For samples accordant with the standard spatial distribution, the ANOVA test was applied (for many groups) and Student’s test (for two groups). In the majority of cases in which the data did not show compliance with the standard distribution, the non-parametric Kruskal–Wallis test was used. A multiple comparison of average ranks for all the samples or the Duncan test were applied as a *post hoc* test. Differences amounting to *P* < 0.05 were considered statistically significant.

The correlation between variables was tested by means of Spearman’s correlation coefficient and multiple regression (Van Emden, 2008). In order to demonstrate statistical differences between the genome size for the examined populations, the one-way analysis of variance (ANOVA) and the *post-hoc* Duncan test were carried out.

## Results

### Morphological variability of shoots

The height of *E. helleborine* shoots ranged from 17.0 to 149.0 cm for the anthropogenic populations and from 4.4 to 95.0 cm for the natural populations. The highest shoot length was 149.0 cm, recorded in the A1 (Guszczewina) population, and the shortest was 4.4 cm, recorded in the N1 population (Góry Kaczawskie); Table S3. In the case of the anthropogenic populations, *E. helleborine* shoots were longer than shoots from the natural populations. In contrast, the mean values of the remaining parameters (i.e., inflorescence length, leaf width and length) were higher in the populations from natural habitats (Table 2). Intra-population variability was demonstrated in terms of all *E. helleborine* features; however, the arrangement of the homogeneous populations investigated during the first period of observation was not repeated in the next period. The greatest intra-population diversity was indicated for the length of shoots, where four homogeneous groups were observed (Table 2). The length of shoots (HS) in the anthropogenic populations demonstrated insignificant variation, ranging from 21.0 to 39.7%, while for the natural populations it ranged from 21.4 to 51.9% (Table 2). The length of inflorescence (LI) demonstrated the highest variation both for the anthropogenic and natural populations. For intra-habitat variation, statistically significant differences in the two investigated periods were demonstrated for the length of shoots, inflorescence and leaves.

**Table 2:** Variation of morphological features of generative *E. helleborine* shoots in analyzed populations during two successive years 2011–2012. Homogeneous letters indicate homogeneous groups (Kruskala–Wallisa test *P* = 0.05). HS, height of shoot; LI, length of inflorescences; WL, width of leaf; LL, length of leaf; CV, coefficient of variation (%). Characters abbreviated of populations as in the Table 1.

Population	HS (cm)		CV%	LI (cm)		CV%	WL (cm)		CV%	LL (cm)		CV%
2011												
A1	84.8	a	28.8	20.9	a	43.2	4.8	a	27.4	8.8	a	27.4
A2	54.1	b	29.3	14.2	b	51.1	4.6	a	34.0	9.0	a	31.1
A3	56.3	b	21.0	11.5	c	46.4	3.1	b	42.8	6.1	b	39.1
A4	42.0	c	30.1	7.0	d	69.0	3.6	b	29.4	7.8	b	23.7
Mean	**59.3**			**13.4**			**4.0**			**8.9**		
N1	62.0	b	28.9	15.8	b	54.0	6.0	c	38.6	11.6	c	19.7
N2	57.4	b	27.9	15.8	b	41.1	5.0	a	32.0	9.8	a	19.8
N3	34.5	d	44.2	11.7	c	53.2	4.2	a	32.2	5.2	d	63.1
N4	48.4	c	48.8	17.0	b	53.3	3.8	b	37.6	6.6	b	33.5
Mean	**50.6**			**15.1**			**5.0**			**8.1**		
2012												
A1	87.7	a	25.7	19.3	a	39.5	6.0	a	25.1	13.2	a	17.7
A2	64.3	b	30.3	18.5	a	51.6	5.8	a	28.0	10.0	b	20.6
A3	56.0	b	34.1	10.2	b	55.2	3.3	b	45.6	7.7	c	34.9
A4	40.2	c	39.7	7.9	b	50.4	3.6	b	26.5	7.7	c	22.9
Mean	**62.1**			**14.0**			**6.8**			**7.5**		
N1	66.7	b	20.4	24.5	c	35.3	5.7	a	27.0	11.5	b	27.0
N2	62.1	b	26.7	16.2	a	52.0	6.0	a	39.3	11.7	b	19.8
N3	55.2	b	36.8	19.6	a	50.4	5.2	a	35.6	10.0	b	32.9
N4	46.1	c	51.9	17.1	a	53.6	3.8	b	37.4	6.6	c	33.5
Mean	**57.5**			**19.4**			**5.2**			**10.0**		

### Morphological variability of flowers

The mean values of the measured elements of the analysed flowers indicated that the flowers from the studied anthropogenic habitats were bigger than the flowers from the natural habitats (*t*-Student’s test, *P* < 0.05; Table 3).

**Table 3:** Biometric characteristics of *E. helleborine* flower in analyzed habitats. Abbreviations as in Table S1.

Feature	Anthropogenic habitat	CV (%)	Natural habitat	CV (%)	*t*-Student’s test (*P* < 0.05)
(mm^2^)					
ALP	36.8	19.4	31.9	28.3	ns
AMS	41.7	26.6	36.0	28.9	ns
ARP	36.0.	19.8	32.5	27.2	ns
ALS	43.2	20.2	34.9	27.2	* (*t* = 2.741, *P* = 0.007)
ARS	42.9	19.9	35.3	29.8	* (*t* = 3.317, *P* = 0.001)
AH	14.9	23.9	12.4	32.8	ns
AE	16.4	19.2	13.5	24.6	* (*t* = 3.060, *P* = 0.003)
(mm)					
CLP	28.2	8.8	25.3	13.3	* (*t* = 3.504, *P* = 0.000)
CMS	29.8	11.3	27.8	13.9	ns
CRP	27.5	9.2	25.9	11.5	* (*t* = 2.060, *P* = 0.003)
CLS	32.3	9.5	28.3	14.1	* (*t* = 4.138, *P* = 0.000)
CRS	31.9	8.4	28.3	16.5	* (*t* = 3.230, *P* = 0.001)
CH	15.4	11.0	13.3	16.3	* (*t* = 4.102, *P* = 0.000)
CE	19.5	9.0	15.9	12.6	* (*t* = 3.350, *P* = 0.001)
(mm)					
LLi	8.1	12.0	7.1	13.0	* (*t* = 4.90, *P* = 0.000)
LLP	10.4	10.4	9.3	13.0	ns
LMS	11.1	12.9	10.9	14.7	ns
LRP	10.1	12.0	9.5	11.9	ns
LLS	12.1	13.6	10.6	17.1	ns
LRS	11.7	10.7	10.7	15.6	ns
(mm)					
WLP	5.2	13.3	5.2	14.1	ns
WMS	5.1	14.7	4.8	17.6	ns
WRP	5.3	18.1	5.3	15.6	ns
WLS	5.4	22.0	4.8	17.0	ns
WRS	5.3	11.6	5.0	14.4	ns

**Notes.**

∗*P* < 0.05 significance level ns non-significant CV coefficient of variation (%)

The analysed flowers differed significantly (*t*-test, *P* < 0.05) in terms of ten biometric features (ALS, ARS, AE, CLP, CRP, CLS, CRS, CH, CE, LLi; Table 3). The biggest differences were observed in the surface area of their perianth petals and sepals. The plants in the studied anthropogenic habitats had evolved flowers in which the surface of the left petal and the right sepal was greater than for the flowers from the studied natural habitats (43.3 mm^2^ and 42.9 mm^2^ in the analysed anthropogenic habitats (A1–A4), while in the natural habitats—34.8 and 35.3 mm^2^ (N1–N4) respectively) (Table 4). The coefficient of variation was higher for the parts of flowers in the analysed anthropogenic habitats (Table 3). The highest value of the coefficient of variation for both habitats was connected with the area of the measured elements, while the smallest value with the perianth perimeter, as well as the perimeter and the length of labellum.

**Table 4:** Biometric characteristic of traits of *E. helleborine* flower in analyzed populations. (*x*, arithmetic mean, F, value of *F* test, *P*, significance level). Abbreviations as in Table S1.

Feature	A1	A2	A3	A4	Average	N1	N2	N3	N4	*x*	*F*	*P*
(mm^2^)												
ALP	35.6	33.7	38.4	39.6	36.8	31.9	32.9	27.0	35.6	31.9	1.8	0.0996
AMS	38.1	40.0	38.7	49.7	41.7	34.7	39.0	30.5	40.0	36.0	1.4	0.2198
ARP	33.7	32.9	39.4	37.9	36.0	33.2	34.1	27.7	34.9	32.5	1.5	0.1753
ALS	42.3	42.0	44.0	44.8	43.3	37.8	35.1	29.7	36.8	34.8	2.4	0.0288
ARS	39.8	42.5	42.3	47.0	42.9	36.5	36.8	30.6	37.3	35.3	1.9	0.0918
AH	13.1	14.0	15.8	16.8	14.9	10.0	18.1	11.3	10.4	12.4	0.8	0.6301
AE	14.4	15.5	17.7	17.8	16.4	13.9	14.7	12.3	13.1	13.5	2.7	0.0185
(mm)												
CLP	27.7	27.2	28.8	28.9	28.2	25.6	25.7	23.7	26.3	25.3	2.6	0.0212
CMS	28.7	29.3	29.4	31.7	29.8	28.6	28.7	25.3	28.7	27.8	1.8	0.1014
CRP	27.5	26.4	28.6	27.8	27.5	26.4	26.3	24.1	26.7	25.9	1.8	0.0974
CLS	31.3	32.0	32.9	33.3	32.3	29.6	28.2	26.1	29.4	28.3	3.7	0.0024
CRS	29.9	31.9	32.3	33.3	31.7	29.1	28.8	26.6	28.9	28.4	2.2	0.044
CH	14.1	15.1	16.0	16.4	15.4	12.8	13.5	13.9	13.0	13.3	3.7	0.0023
CE	16.4	17.0	18.7	18.3	17.6	16.2	16.2	15.3	16.0	15.9	2.9	0.0108
LLi	7.7	8.0	8.3	8.5	8.1	6.9	7.5	6.9	7.1	7.1	3.2	0.0063
LLP	10.4	10.2	10.6	10.6	10.5	8.5	9.4	9.3	9.4	9.2	1.3	0.2808
LMS	5.2	5.0	5.4	5.6	5.3	4.7	5.6	5.1	5.1	5.1	0.6	0.7426
LRP	11.1	11.0	11.4	11.7	11.3	9.7	11.0	11.0	11.3	10.7	1.4	0.2208
LLS	4.9	5.2	4.8	6.0	5.2	4.5	5.0	4.9	4.8	4.8	0.6	0.7848
LRS	10.4	9.8	10.2	10.3	10.2	8.6	9.8	9.4	9.9	9.5	0.9	0.5069
WLP	5.5	5.0	5.6	5.5	5.4	4.8	5.4	5.5	5.1	5.2	0.8	0.5561
WMS	11.9	12.0	12.3	12.2	12.1	9.4	11.0	11.0	10.4	10.5	2.5	0.0244
WRP	5.5	5.3	5.3	5.5	5.4	4.7	4.8	5.0	4.8	4.8	1.2	0.3090
WLS	11.4	11.9	11.6	12.6	11.9	9.8	11.0	10.5	10.9	10.6	1.5	0.1840
WRS	5.1	5.3	5.3	5.7	5.3	4.7	4.9	5.2	4.8	4.9	0.6	0.7912

The correlation analysis of the metric features of the flowers in both types of habitats pointed to a strong correlation (*r* > 0.90) between the surface and the perimeter of perianth petals and sepals (Tables S1, S2). The flowers from the studied anthropogenic habitats demonstrated a very strong correlation between the labellum length and the surface perimeter of hypochile and epichile. However, the correlation between the same features in the flowers from the studied natural habitats varied from moderate to strong. The correlations between the epichile and hypochile features for the flowers of the studied anthropogenic habitats were strong or very strong, while for the flowers in the analysed natural habitats weak to moderate. The conducted multiple regression analysis revealed a strong correlation between the perimeter of epichile and the labellum length (*r*^2^ = 0.73) in the studied anthropogenic populations. In the flowers from the studied natural habitats, the correlations between the length of labellum and epichile area, the left and right petals, and left sepal as well as the perimeter of left and right petals were found.

### Genome size estimation

**Figure 3:**
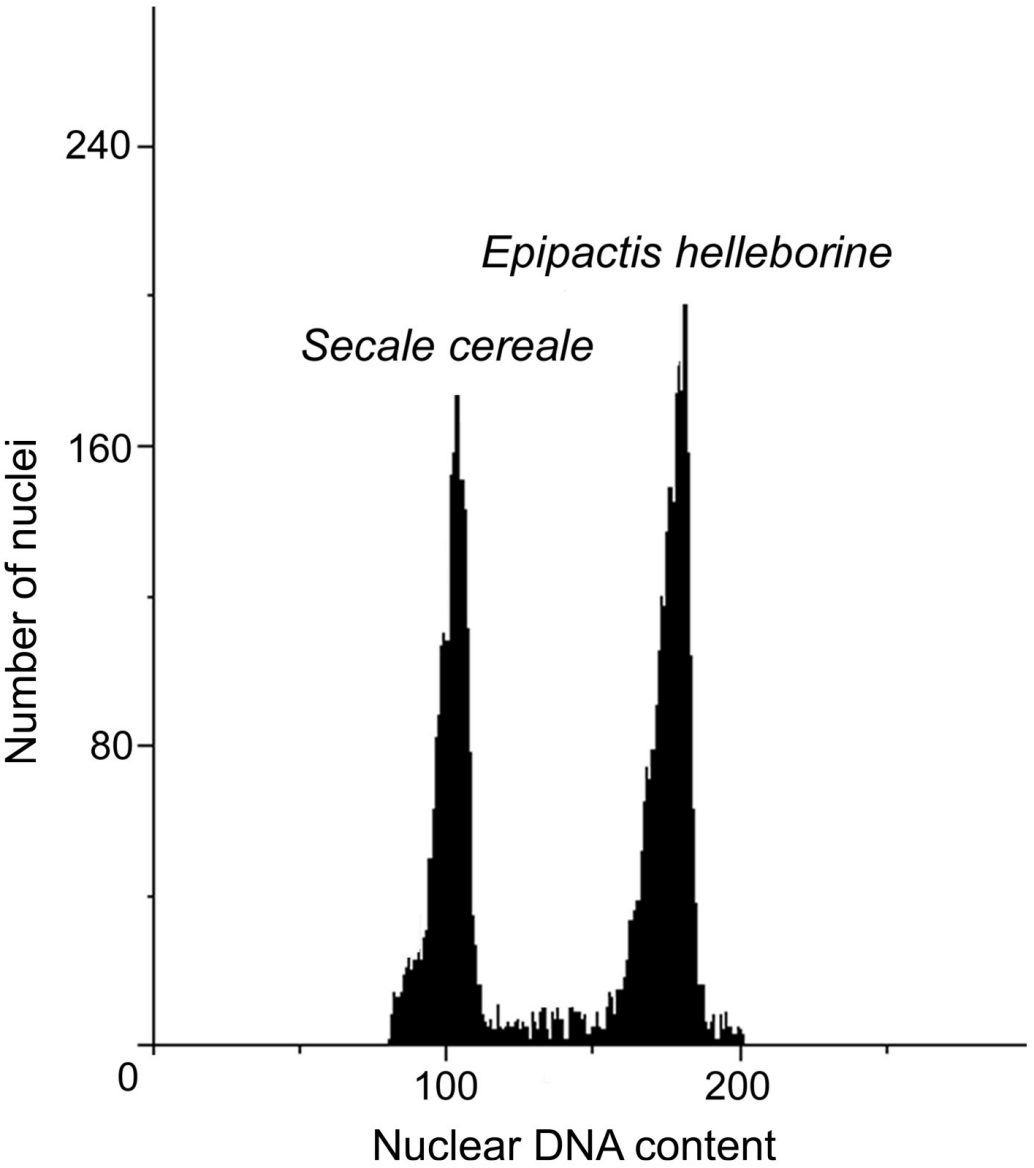
Histograms of nuclear DNA contents of *E. helleborine*.

The DNA content values of the analysed accessions from natural habitats ranged from 27.32 (N2 and N3) to 27.89 pg/2C. The anthropogenic populations resulted in the DNA content range from 27.49 (A4) to 28.39 pg/2C (A3). Within analysed populations, both natural and anthropogenic, two populations with increased DNA content could be distinguished: A3 with the highest DNA content (28.39 pg/2C) and N4 (27.89 pg/2C). The genome size of A3 population were about 1 pg higher than genome size of other anthropogenic populations. Also one population (N4) from natural environment revealed about 0.5 pg/2C higher value of genome size than other plants in natural populations (Table 5, Fig. 3).

**Table 5:** Genome size of *E. helleborine* plants in the anthropogenic (A) and natural (N) populations. Homogeneous letters indicate homogeneous groups.

Population	2C DNA content [pg ± SD]	
A1	27.57 ± 0.239	c
A2	27.49 ± 0.166	c
A3	28.39 ± 0.279	a
A4	27.39 ± 0.348	c
Mean	**27.71**	
N1	27.42 ± 0.072	c
N2	27.32 ± 0.088	c
N3	27.32 ± 0.168	c
N4	27.89 ± 0.159	b
Mean	**27.49**	

## Discussion

### The height of shoots

Phenotypic plasticity of the species is an expression of its potential abilities to colonise areas which differ in terms of many habitat features (Sultan, 1995; Sultan, 2000; Sultan, 2001). A response of a plant to environmental conditions can manifest itself in its morphological variability, physiological responses or reproductive potential.

Species from the genus *Epipactis* belong to a group with highly variable phenotype features (Rewicz et al., 2017a; Rewicz et al., 2017b). Flower and seed features, as well as the arrangement of leaves, are the elements least susceptible to environmental changes (Heywood, 1974), and therefore are used in taxonomy. In contrast, the features most vulnerable to environmental changes are the height of shoots, the leaf size, the colour and size of flower and the length of flowering period (Ehlers, Olesen & Gren, 2002; Jakubska-Busse, 2008; Stace, 1993; Sultan, 1995). The shoot length, the length of inflorescence and the leaf size have proven to be the most variable characteristics, and therefore the results support the view that such features are the most susceptible to environmental changes (Heslop-Harrison, 1953; Heywood, 1974). The literature data on the length of the *E. helleborine* shoot earlier reported indicate it was within the range of 18.0–100.0 cm (Bernardos, Amich & Crespi, 2003; Delforge, 2006; Harrap & Harrap, 2010; Hegi, 1925). The mean length of generative shoots for the examined anthropogenic populations ranged from 59.3 to 62.1 cm, while for the populations from the studied natural habitats from 50.6 to 57.5 cm. Overall, the average length of shoots from the populations in the analysed anthropogenic habitats was higher than in the natural populations. Moreover, studies of other authors confirm considerable variability of this particular feature (Adamowski, 2006; Bîtea et al., 2011). The maximum and minimum length of *E. helleborine* shoots in the examined anthropogenic habitats ranged from 17.0 to 149.0 cm, while in the natural populations from 4.4 to 95.0 cm. Keller & Schlechter (1928) found shoots from 30.0 to 125.0 cm long, while Adamowski (2006) reported 130.0 cm long shoots growing on a poplar plantation. Also, Solarz (1994) found 120.0 cm long shoots on a narrow-gauge railway embankment and 103.0 cm long shoots in the population growing in a pine forest. It is believed that light is one of the most vital environmental stimuli determining phenotypic plasticity (Herman & Sultan, 2011). The highest shoots with the longest inflorescence and the largest leaves were recorded in the anthropogenic populations in Guszczewina (A1) and Hajnówka (A2). The shoots in those populations grew in the full sun, without shade from trees and bushes. The remaining anthropogenic populations grew in partial shade. Part of the Sulejów 1 (A3) population grew in the pine forest of *Peucedano-Pinetum*, while the Sulejów 2 (A4) population in the ruderal poplar thicket of *Populus* sp., *Acer platanoides* and *Robinia pseudoacacia* saplings. In those populations, generative shoots were shorter in comparison to the shoots from the A1 and A2 populations. On the other hand, all the *E. helleborine* shoots from the populations in the studied natural habitats grew under the canopy of trees and the height of their generative shoots ranged from 34.5 (the N3 population) to 66.7 cm (the N1 population).

According to Harper (1986), plants at new sites often achieve considerable sizes in accordance with the strategy of “race to the sun”. This study has also confirmed that in the examined anthropogenic habitats the “plant-plant” interaction occurred. Therefore, the shoots in close vicinity are similar in terms of height and shape to the ones observed particularly in the A1 and A2 populations. Adamowski (2006) suggests that the occurrence of high *E. helleborine* ramet is also influenced by the presence of species from the genus *Populus* sp. This is connected with the phenomenon of mycorrhiza occurring between fungus poplar and *E. helleborine* (McCormick, Whigham & O’Neill, 2004). Our results have not confirmed unequivocally the correlation between *E. helleborine* and poplar since in the A1 and A2 populations with the highest shoots such trees were not present. However, in the A4 population, where *E. helleborine* grew among *Populus* ×* canadensis*, the shoots were shorter in comparison to those from the A1 and A2 populations.

In the case of leaf size in both habitats, the leaf length ranged from 1.7 cm to 18.0 cm and the leaf width from 1.5 to 13.0 cm. The results obtained in this study differ significantly from values found in the literature, where *E. helleborine* leaf length ranged from 4.0 to 13.0 cm, while the leaf width from 2.0 to 7.0 cm (Delforge, 2006; Bîtea et al., 2011). High variability of leaf size has confirmed the findings of other authors (Jakubska-Busse et al., 2016; Guo et al., 2007; Navas & Garniere, 2002) that the leaf size is affected by environmental stresses to which plants react by changing the size of their leaves. Populations growing in anthropogenic habitats are certainly subjected to constant and rapid environmental changes (like water relations, air pollution, soil pollution). The variability of leaf width and length dependent on environmental conditions, particularly their correlation with light, is confirmed by studies carried out by Xu et al. (2008) on *Quercus acutissima* Carruth. and Pandey & Nagar (2002) on the phenotypic variability of *Valeriana jatamansi* Jones ex Roxb*.*

### Perianth features

Flowers from the studied anthropogenic habitats were also higher than those from the analysed natural habitats. Therefore, the conclusion might be drawn that the labellum is a part of the perianth which is not “sufficiently” resistant to environmental changes, as shown by statistically insignificant differences in the elements recorded in the analysed habitats. This is also confirmed by the results of Ehlers, Olesen & Gren (2002) who revealed high variability of the epichile length, as well as the length and width of perianth petals (Hegi, 1925; Delforge, 2006). Also, there are no data in the literature concerning the surface and the perimeter of the *E. helleborine* perianth*.*

The differences which were revealed in morphology of *E. helleborine* flower as well as the data concerning leaf morphology have enhanced and complemented the number of features influencing the phenotypic plasticity of the taxon. These differences do not support Falińska’s (1974) opinion that modifications of morphological characteristics of the shoot-ground, as a sign of adaptation to the particular environmental parameters where the plant exists, are usually not revealed in the structure of its generative organs.

Despite the indicated high phenotypic plasticity of the species, the plants growing on roadsides in the examined anthropogenic habitats were not polyploids. This is confirmed by studies carried out by other researchers, for instance, Bernardos, Amich & Crespi (2003), who revealed that *E. helleborine* growing in different habitats were diploid.

A broad range of morphological variability (manifested by particularly splendid ramets in the absence of polyploid specimens) of the examined specimens observed in all the studied populations may be a result of implementing the epistasis model (Scheiner, 1993). It assumes that plasticity is evoked by genes determining the amount of phenotypic response to the impact of environment. In our opinion, variations between populations growing in natural and anthropogenic habitats (e.g., roadside) are phenotypic plasticity which are not supported at a genetic level. This is influenced by dynamic changes of habitat conditions, such as shading water conditions or the composition of accompanying specimens (Doust & Doust, 1988).

The genome size of *E. helleborine* obtained in this study is slightly higher than reported earlier by Prat, Brown & Gevandan (2014; about 25.5 pg/2C). This can be explained by different buffer and internal standard used. Nevertheless, according to Soltis et al. (2003), *E. helleborine* can be classified into the group of plants with intermediate genomes (<14 pg/1C; mean for all populations). The genome size of the most of the investigated populations was homogenous, however one natural (N4) and one anthropogenic (A3) population differed in this trait. Increase in DNA content of those populations could be explained by the occurrence of an aneuploidy since different chromosome numbers were observed for this species (Silvestre, 1983). The changes in genome size occurring both in natural and anthropogenic populations rather excluded impact of environment on genome size of *E. helleborine*. In our opinion, the obtained results may form a basis for a new study distinguishing *E. helleborine* ecofens, which is particularly well-grounded in the case of very large *E. helleborine* specimens occupying anthropogenic habitats.

According to some researchers, the presence of phenotypic plasticity and the occurrence of ecotypes are adaptation strategies of plants to environmental changes (Jacquemyn et al., 2018; Stace, 1993). In our opinion, variations between populations growing in natural and anthropogenic habitats (roadside) are phenotypic plasticity which are not supported by a genetic level. But on the populations from anthropogenic habitats we reported the set of characteristic features, i.e., high shoots, long inflorescence and long, broad leaves which may indicate the occurrence of ecofen. We agree, however, that it is difficult to isolate a taxonomic unit for ecofen due to the lack of experimental research and molecular investigation.

##  Supplemental Information

10.7717/peerj.5992/supp-1 Table S1 List of abbreviations of studied features of *E. helleborine*

10.7717/peerj.5992/supp-2 Table S2 Values of correlation (Spearman coefficient) for studied morphological traits of *Epipactis helleborine* Values in bold are significant for *p* < 0.05. Abbreviations as in Table 1.

10.7717/peerj.5992/supp-3 Table S3 Minimum and maximum values of *E. helleborine* shoots

10.7717/peerj.5992/supp-4 Supplemental Information 1 Flower measurements (raw data)

## Additional Information and Declarations

### Field Study Permissions

The following information was supplied relating to field study approvals (i.e., approving body and any reference numbers): Field experiments were approved by the Regional Directoriate of Environmental Protection in Białystok (Regionalna Dyrekcja Ochrony Środowiska w Białymstoku) (permit WPN6400.74.2013.MW).

### Data Availability

The following information was supplied regarding data availability: The raw data are provided in Supplemental Files.
