# Supplementary material for: Morphology and genome size of Epipactis helleborine (L.) Crantz (Orchidaceae) growing in anthropogenic and natural habitats
Source: PeerJ. 2018 Dec 20;6:e5992. doi: 10.7717/peerj.5992 (PMC6304265; doi:10.7717/peerj.5992)
Supplement: Table S1 [file peerj-06-5992-s001.doc]

Supplementary Table S1. List of abbreviations of studied features of *E. helleborine.*

| Code | Description |
| --- | --- |
| Abbreviations of traits. Part A | |
| A | Area |
| C | Circuit |
| L | Length |
| W | Width |
| Abbreviations of flower elements. Part B | |
| MS | Middle Sepal |
| LP | Left Petal |
| RP | Right Petal |
| LS | Left Sepal |
| RS | Right Sepal |
| E | Epichile |
| H | Hypochile |
| Combine abbreviations of features. Part C=Part A + B | |
| AE | Area of the Epichile (mm^2^) |
| AH | Area of the Hypochile (mm^2^) |
| ALS | Area of the Left Sepal (mm^2^) |
| AMS | Area of the Middle Sepal (mm^2^) |
| ARS | Area of the Right Sepal (mm^2^) |
| ALP | Area of the Left Petal (mm^2^) |
| ARP | Area of the Right Petal (mm^2^) |
| CE | Circuit of the Epochile (mm) |
| CH | Circuit of the Hypochile (mm) |
| CLS | Circuit of the Left Sepal (mm) |
| CMS | Circuit of the Middle Sepal (mm) |
| CRS | Circuit of the Right Sepal (mm) |
| CLP | Circuit of the Left Petal (mm) |
| CRP | Circuit of the Right Petal (mm) |
| LLi | Length of the Lip (mm) |
| LLS | Length of the Left Sepal (mm) |
| LRS | Length of the Right Sepal (mm) |
| LMS | Length of the Middle Sepal (mm) |
| LLP | Length of the Left Petal (mm) |
| LRP | Length of the Right Petal (mm) |
| WRS | Width of the Right Sepal(mm) |
| WLS | Width of the Left Sepal (mm) |
| WMS | Width of the Middle Sepal (mm) |
| WLP | Width of the Left Petal (mm) |
| WRP | Width of the Right Petal (mm) |
