# Supplementary material for: Morphology and genome size of Epipactis helleborine (L.) Crantz (Orchidaceae) growing in anthropogenic and natural habitats
Source: PeerJ. 2018 Dec 20;6:e5992. doi: 10.7717/peerj.5992 (PMC6304265; doi:10.7717/peerj.5992)
Supplement: Table S2 — Values in bold are significant for p < 0.05. Abbreviations as in Table 1. [file peerj-06-5992-s002.docx]

Supplementary Table 2. Values of correlation (Spearman coefficient) for studied morphological traits of *Epipactis helleborine*. Values in bold are significant for p<0.05. Abbreviations as in Table 1

|  | ALP | CLP | AMS | CMS | ARP | CPL | ALS | CLS | ARS | CRS | Lli | AH | CH | AE | CE | LRP | WRP | LMS | WMS | LPRP | WP | LRS | WRS | LLS | WLS |
| --- | --- | --- | --- | --- | --- | --- | --- | --- | --- | --- | --- | --- | --- | --- | --- | --- | --- | --- | --- | --- | --- | --- | --- | --- | --- |
| ALP | 1.00 | 0.90 | 0.73 | 0.77 | 0.85 | 0.79 | 0.73 | 0.63 | 0.76 | 0.70 | 0.41 | 0.50 | 0.44 | 0.45 | 0.57 | 0.78 | 0.90 | 0.66 | 0.74 | 0.55 | 0.60 | 0.54 | 0.72 | 0.50 | 0.34 |
| CLP |  | 1.00 | 0.64 | 0.85 | 0.80 | 0.87 | 0.67 | 0.66 | 0.60 | 0.68 | 0.49 | 0.38 | 0.33 | 0.45 | 0.59 | 0.91 | 0.77 | 0.77 | 0.66 | 0.62 | 0.54 | 0.59 | 0.64 | 0.45 | 0.29 |
| AMS |  |  | 1.00 | 0.75 | 0.66 | 0.59 | 0.63 | 0.58 | 0.61 | 0.60 | 0.44 | 0.38 | 0.28 | 0.59 | 0.62 | 0.67 | 0.55 | 0.69 | 0.61 | 0.48 | 0.43 | 0.58 | 0.56 | 0.42 | 0.33 |
| CMS |  |  |  | 1.00 | 0.78 | 0.86 | 0.70 | 0.76 | 0.60 | 0.71 | 0.48 | 0.27 | 0.14 | 0.59 | 0.67 | 0.77 | 0.67 | 0.95 | 0.65 | 0.66 | 0.47 | 0.77 | 0.54 | 0.48 | 0.29 |
| ARP |  |  |  |  | 1.00 | 0.92 | 0.74 | 0.68 | 0.76 | 0.77 | 0.52 | 0.51 | 0.42 | 0.57 | 0.63 | 0.74 | 0.70 | 0.70 | 0.66 | 0.68 | 0.60 | 0.64 | 0.66 | 0.55 | 0.34 |
| CPL |  |  |  |  |  | 1.00 | 0.67 | 0.72 | 0.61 | 0.70 | 0.48 | 0.33 | 0.25 | 0.54 | 0.62 | 0.81 | 0.68 | 0.80 | 0.61 | 0.73 | 0.55 | 0.68 | 0.56 | 0.47 | 0.31 |
| ALS |  |  |  |  |  |  | 1.00 | 0.89 | 0.89 | 0.92 | 0.24 | 0.34 | 0.27 | 0.47 | 0.46 | 0.65 | 0.58 | 0.68 | 0.56 | 0.58 | 0.36 | 0.82 | 0.87 | 0.70 | 0.25 |
| CLS |  |  |  |  |  |  |  | 1.00 | 0.73 | 0.87 | 0.29 | 0.14 | 0.08 | 0.47 | 0.49 | 0.66 | 0.55 | 0.75 | 0.55 | 0.57 | 0.39 | 0.95 | 0.68 | 0.66 | 0.19 |
| ARS |  |  |  |  |  |  |  |  | 1.00 | 0.91 | 0.28 | 0.45 | 0.38 | 0.46 | 0.45 | 0.55 | 0.61 | 0.56 | 0.57 | 0.54 | 0.38 | 0.65 | 0.82 | 0.65 | 0.35 |
| CRS |  |  |  |  |  |  |  |  |  | 1.00 | 0.31 | 0.30 | 0.26 | 0.48 | 0.47 | 0.66 | 0.55 | 0.70 | 0.54 | 0.63 | 0.35 | 0.81 | 0.81 | 0.71 | 0.30 |
| Lli |  |  |  |  |  |  |  |  |  |  | 1.00 | 0.56 | 0.46 | 0.70 | 0.69 | 0.40 | 0.28 | 0.46 | 0.27 | 0.44 | 0.27 | 0.26 | 0.19 | 0.26 | 0.07 |
| AH |  |  |  |  |  |  |  |  |  |  |  | 1.00 | 0.94 | 0.28 | 0.35 | 0.31 | 0.34 | 0.17 | 0.36 | 0.30 | 0.28 | 0.08 | 0.39 | 0.29 | 0.13 |
| CH |  |  |  |  |  |  |  |  |  |  |  |  | 1.00 | 0.13 | 0.21 | 0.29 | 0.31 | 0.05 | 0.29 | 0.23 | 0.24 | -0.01 | 0.37 | 0.26 | 0.09 |
| AE |  |  |  |  |  |  |  |  |  |  |  |  |  | 1.00 | 0.90 | 0.43 | 0.34 | 0.59 | 0.40 | 0.41 | 0.37 | 0.45 | 0.47 | 0.31 | 0.27 |
| CE |  |  |  |  |  |  |  |  |  |  |  |  |  |  | 1.00 | 0.52 | 0.51 | 0.59 | 0.60 | 0.35 | 0.55 | 0.47 | 0.47 | 0.26 | 0.35 |
| LRP |  |  |  |  |  |  |  |  |  |  |  |  |  |  |  | 1.00 | 0.57 | 0.73 | 0.56 | 0.58 | 0.50 | 0.62 | 0.60 | 0.41 | 0.33 |
| WRP |  |  |  |  |  |  |  |  |  |  |  |  |  |  |  |  | 1.00 | 0.53 | 0.78 | 0.43 | 0.57 | 0.44 | 0.58 | 0.39 | 0.31 |
| LMS |  |  |  |  |  |  |  |  |  |  |  |  |  |  |  |  |  | 1.00 | 0.43 | 0.71 | 0.33 | 0.79 | 0.49 | 0.49 | 0.22 |
| WMS |  |  |  |  |  |  |  |  |  |  |  |  |  |  |  |  |  |  | 1.00 | 0.30 | 0.57 | 0.43 | 0.62 | 0.40 | 0.32 |
| LPRP |  |  |  |  |  |  |  |  |  |  |  |  |  |  |  |  |  |  |  | 1.00 | -0.10 | 0.54 | 0.44 | 0.43 | 0.25 |
| WP |  |  |  |  |  |  |  |  |  |  |  |  |  |  |  |  |  |  |  |  | 1.00 | 0.38 | 0.37 | 0.28 | 0.17 |
| LRS |  |  |  |  |  |  |  |  |  |  |  |  |  |  |  |  |  |  |  |  |  | 1.00 | 0.55 | 0.65 | 0.14 |
| WRS |  |  |  |  |  |  |  |  |  |  |  |  |  |  |  |  |  |  |  |  |  |  | 1.00 | 0.56 | 0.34 |
| LLS |  |  |  |  |  |  |  |  |  |  |  |  |  |  |  |  |  |  |  |  |  |  |  | 1.00 | -0.41 |
| WLS |  |  |  |  |  |  |  |  |  |  |  |  |  |  |  |  |  |  |  |  |  |  |  |  | 1.00 |
