# Supplementary material for: Morphology and genome size of Epipactis helleborine (L.) Crantz (Orchidaceae) growing in anthropogenic and natural habitats
Source: PeerJ. 2018 Dec 20;6:e5992. doi: 10.7717/peerj.5992 (PMC6304265; doi:10.7717/peerj.5992)
Supplement: Table S3 [file peerj-06-5992-s003.docx]

| Population | HS (cm) | LI (cm) | WL (cm) | LL (cm) |
| --- | --- | --- | --- | --- |
| 2011 | | | | |
| A1 | 42.0-149.0 | 2.0-36.0 | 1.0-7.0 | 4.0-13.0 |
| A2 | 31.0-90.0 | 2.0-33.0 | 1.5-8.0 | 3.0-13.0 |
| A3 | 40.0-80.0 | 3.0-27.0 | 1.2-7.0 | 2.5-11.0 |
| A4 | 20.0-80.0 | 1.0-22.0 | 1.5-5.9 | 5.0-12.0 |
| N1 | 32.0-90.0 | 5.0-37.0 | 2.8-13.0 | 7.2-15.5 |
| N2 | 17.0-99.0 | 4.0-31.0 | 1.5-8.5 | 5.6-13.5 |
| N3 | 4.4-60.0 | 2.0-27.5 | 1.7-9.0 | 1.**7-**10.5 |
| N4 | 16.0-95.0 | 4.0-40.0 | 1.8-7.4 | 3.5-12.6 |
| 2012 | | | | |
| A1 | 30.0-129.0 | 6.0-41.0 | 3.3-11.0 | 8.0**-**18.0 |
| A2 | 26.0-107.0 | 2.0-36.0 | 3.0-10.0 | 6.0-15.0 |
| A3 | 21.0-90.0 | 2.0-21.0 | 1.4-5.7 | 3.2-11.5 |
| A4 | 17.0-78.0 | 1.0-20.0 | 2.0-5.9 | 5.0-12.0 |
| N1 | 42.0-85.0 | 4.5-35.0 | 2.5-7.9 | 6.5-17.5 |
| N2 | 34.0-90.0 | 5.0-37.0 | 2.8-13.0 | 7.2-15.5 |
| N3 | 9.4-85.0 | 4.0-35.0 | 1.77.9 | 5.0-17.5 |
| N4 | 7.6-95.0 | 4.0-40.0 | 1.8-7.6 | 3.5-12.6 |
